# Filamentous Bacteriophages and the Competitive Interaction between Pseudomonas aeruginosa Strains under Antibiotic Treatment: a Modeling Study

**DOI:** 10.1128/mSystems.00193-21

**Published:** 2021-06-22

**Authors:** Julie D. Pourtois, Michael J. Kratochvil, Qingquan Chen, Naomi L. Haddock, Elizabeth B. Burgener, Giulio A. De Leo, Paul L. Bollyky

**Affiliations:** a Department of Biology, Stanford University, Stanford, California, USA; b Hopkins Marine Station, Stanford University, Pacific Grove, California, USA; c Department of Materials Science and Engineering, Stanford University, Stanford, California, USA; d Division of Infectious Diseases and Geographic Medicine, Department of Medicine, Stanford University School of Medicine, Stanford, California, USA; e Center for Excellence in Pulmonary Biology, Department of Pediatrics, Stanford University, Stanford, California, USA; Northern Arizona University; The Ohio State University

**Keywords:** bacteria, filamentous phages, antibiotics, cystic fibrosis, biofilms

## Abstract

Pseudomonas aeruginosa (*Pa*) is a major bacterial pathogen responsible for chronic lung infections in cystic fibrosis patients. Recent work has implicated Pf bacteriophages, nonlytic filamentous viruses produced by *Pa*, in the chronicity and severity of *Pa* infections. Pf phages act as structural elements in *Pa* biofilms and sequester aerosolized antibiotics, thereby contributing to antibiotic tolerance. Consistent with a selective advantage in this setting, the prevalence of Pf-positive (Pf+) bacteria increases over time in these patients. However, the production of Pf phages comes at a metabolic cost to bacteria, such that Pf+ strains grow more slowly than Pf-negative (Pf−) strains *in vitro*. Here, we use a mathematical model to investigate how these competing pressures might influence the relative abundance of Pf+ versus Pf− strains in different settings. Our model suggests that Pf+ strains of *Pa* cannot outcompete Pf− strains if the benefits of phage production falls onto both Pf+ and Pf− strains for a majority of parameter combinations. Further, phage production leads to a net positive gain in fitness only at antibiotic concentrations slightly above the MIC (i.e., concentrations for which the benefits of antibiotic sequestration outweigh the metabolic cost of phage production) but which are not lethal for Pf+ strains. As a result, our model suggests that frequent administration of intermediate doses of antibiotics with low decay rates and high killing rates favors Pf+ over Pf− strains. These models inform our understanding of the ecology of Pf phages and suggest potential treatment strategies for Pf+ *Pa* infections.

**IMPORTANCE** Filamentous phages are a frontier in bacterial pathogenesis, but the impact of these phages on bacterial fitness is unclear. In particular, Pf phages produced by *Pa* promote antibiotic tolerance but are metabolically expensive to produce, suggesting that competing pressures may influence the prevalence of Pf+ versus Pf− strains of *Pa* in different settings. Our results identify conditions likely to favor Pf+ strains and thus antibiotic tolerance. This study contributes to a better understanding of the unique ecology of filamentous phages in both environmental and clinical settings and may facilitate improved treatment strategies for combating antibiotic tolerance.

## INTRODUCTION

Pseudomonas aeruginosa (*Pa*) infections, particularly those that are antibiotic resistant or antibiotic tolerant, are responsible for growing health care expenses and considerable mortality (1). *Pa* infections are particularly problematic in cystic fibrosis (CF), an inherited disease associated with thick, tenacious airway secretions (2–5). The establishment of a chronic *Pa* infection often occurs early in life and evolves into an entrenched and highly damaging condition in adult CF patients. By adulthood, nearly 60% of CF patients have chronic *Pa* infections (6) with 10% of these infections harboring antibiotic-resistant *Pa* strains (7, 8). Understanding the forces that influence the progression of these infections is critical for developing effective clinical treatments.

*Pa* is particularly pathogenic because of its ability to form robust biofilms. Biofilms are viscous conglomerates of polymers and microbial communities that allow *Pa* to colonize airways (9). Once *Pa* biofilm infections are established in CF lungs, they are nearly impossible to eradicate (10, 11). Many antibiotics have limited penetration through biofilms (12) such that the bacteria encased within are antibiotic tolerant (i.e., able to survive exposure to antimicrobials) in comparison to planktonic bacteria (13, 14). Over time, this reduction in effective antimicrobial activity favors the development of antibiotic resistance (13, 15–17) (i.e., the ability to proliferate despite the presence of antibiotics [13]).

The infection of *Pa* strains by Pf phages further contributes to the disease burden of *Pa* in humans and its pathogenicity (18, 19). Pf phages are filamentous, single-stranded DNA viruses in the genus *Inovirus.* Unlike many bacteriophages that lyse their bacterial hosts, Pf phages are produced without lysis (20) and are reported to contribute to *Pa* virulence (21). Many (between 36 and 61%) *Pa* clinical isolates produce Pf phages (7, 22, 23), and the presence of these phages in the sputum of CF patients is associated with the chronicity of *Pa* infections (7, 24) and larger declines in pulmonary function during exacerbation (7, 25, 26). Pf phages likewise enhance the virulence of *Pa* infections in animal models (24), including in airway infections (7).

Among other effects on the bacterial and human hosts, Pf phages affect *Pa* survival through their influence on biofilm formation and function (7, 24, 27). Pf phages promote the ordering of biofilm polymers into a liquid crystalline structure that prevents antibiotic diffusion and inhibits bacterial clearance (18, 26, 28). Some antibiotics are bound to Pf phage structures and are prevented from killing embedded bacteria (24, 29). This liquid crystalline organization has also been observed in other filamentous phages (30). Antibiotic sequestration by these structures could provide an advantage to *Pa*, as most patients with CF are maintained on cycled courses of inhaled, antipseudomonal antibiotics, such as tobramycin and aztreonam (31).

Consistent with a selective advantage in CF lungs, the prevalence of Pf phages increases with patient age and disease severity. Less than 10% of children with CF who are infected with *Pa* have Pf-positive (Pf+) strains, while over 40% of adults and 100% of 10 prelung transplant patients have Pf+ strains (7, 26). The prevalence of Pf phages likewise increases over time in patients with chronic *Pa* wound infections (32). Of note, in a longitudinal cohort study of patients with CF, the acquisition of Pf phage represented the appearance of a new strain of *Pa*. There were no *de novo* acquisition of Pf phages by an existing, unparasitized strain of *Pa* (unpublished results). This suggests that infection of Pf-negative (Pf−) strains from a Pf+ strain is rare. Together these data point to selective advantages for Pf+ *Pa* strains in the setting of chronic infection. However, the production of Pf phages comes at a steep metabolic cost to the *Pa* strains that produce them. Pf phages are abundantly expressed in Pf+ *Pa* biofilms (33) and CF sputum at an average of 10^7^ copies/ml (26, 28). Pf+ strains of *Pa* consequently grow more slowly due to the energetic demands of phage production (18, 21, 26, 34). The impact of these competing pressures on *Pa* fitness is therefore unclear. Mathematical models can yield important insights into these dynamics (35–40).

**FIG 1:**
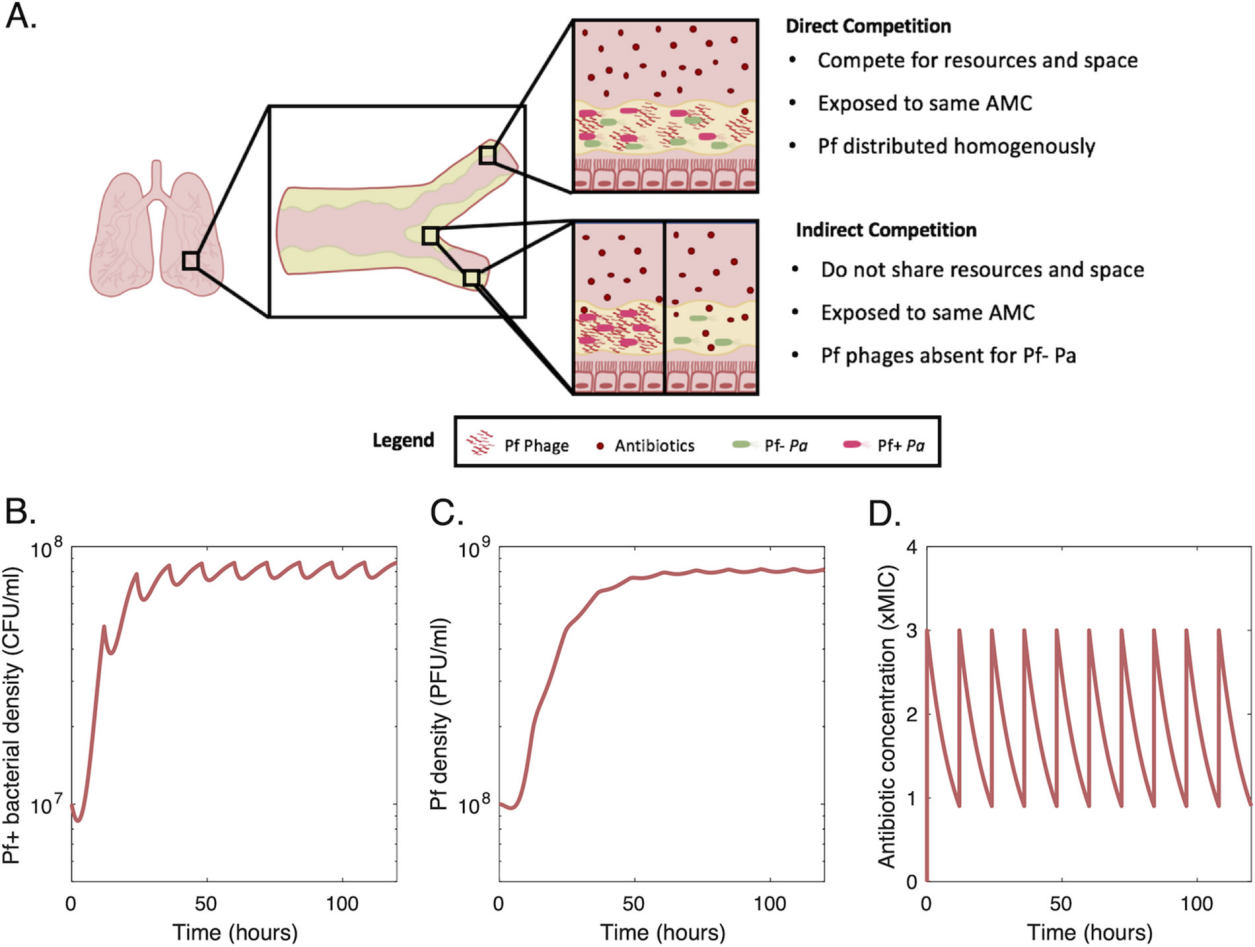
Modeling of the different competitive relationships between Pf+ and Pf− P. aeruginosa (*Pa*) bacterial strains. (A) Strains can either compete directly (i.e., when the strains are colocalized), or indirectly (i.e., the strains are not colocalized, but within the same tissue). In both cases, the antimicrobial concentration (AMC) during treatment is the same. However, sequestration of antibiotic by phages leads to a lower concentration of free antibiotics capable of inducing bacterial cell death. Our model assumes that the lowering of free antibiotic by sequestration benefits any *Pa* bacterial cells within the location, regardless of whether that cell produces Pf phages. (B) Example of the dynamics of a Pf+ bacterial strain and Pf phages (C), over 5 days, with antibiotics delivered twice a day (D).

The microbial ecology of the lung is an important variable in clinical outcomes in CF (41, 42). In addition, filamentous phages also infect a wide range of other bacteria, from Escherichia coli to Vibrio cholerae, affecting their fitness through similarly diverse mechanisms (43). A better understanding of the effect of antibiotic sequestration by phages could shed light on the impact of antibiotics on phage-infected bacteria, in both clinical and environmental settings. In this study, we use a mathematical model to investigate how antibiotic sequestration by phages impacts the competitive dynamics of Pf+ and Pf− strains of *Pa*. Building up on previous modeling studies and experimental results (18, 38, 44), our model assumes that Pf− P. aeruginosa exhibits logistic growth in the absence of antibiotics, that phage production has a metabolic cost causing lower growth of Pf+ *Pa* with respect to Pf− *Pa*, and yet, that under antibiotic treatment, both Pf+ *Pa*’s lower growth and antibiotic sequestration by the phage biofilm might decrease antibiotic impact on Pf+ *Pa* and provide a competitive advantage with respect to Pf− *Pa*. We analyzed these hypotheses under alternative assumptions on direct or indirect interaction between Pf+ and Pf− strains of *Pa* and a realistic range of model parameters according to the results of *in vitro* experiments, clinical studies, and published literature and explore implications for disease treatment (Fig. 1). While general guidelines of inhaled antibiotic therapy in CF do exist, the particular regimen is decided by the provider on a case-by-case basis with mixed clinical success (45–47). We thus use our model to compare the fitness of Pf+ and Pf− strains when exposed to different concentrations comparable to the concentrations of tobramycin in CF lungs and for different metabolic costs. In addition, we investigate how antibiotic characteristics affect this comparison. Although the results of our modeling analysis should not be considered prescriptive, our data suggest that Pf production is most advantageous when bacteria are continuously exposed to intermediate concentrations of antibiotics, consistent with the type of environment created by treatments of chronic bacterial infections, and ecological systems in which bacteria produce antibacterial compounds to prevent competition (48).

## RESULTS

### Fitness of Pf+ and Pf− strains in the absence of antibiotic treatment.

In the absence of antibiotics, Pf− strains reach higher densities than Pf+ strains. They also drive Pf+ strains to extinction when they coexist in the same infection sites.

**FIG 2:**
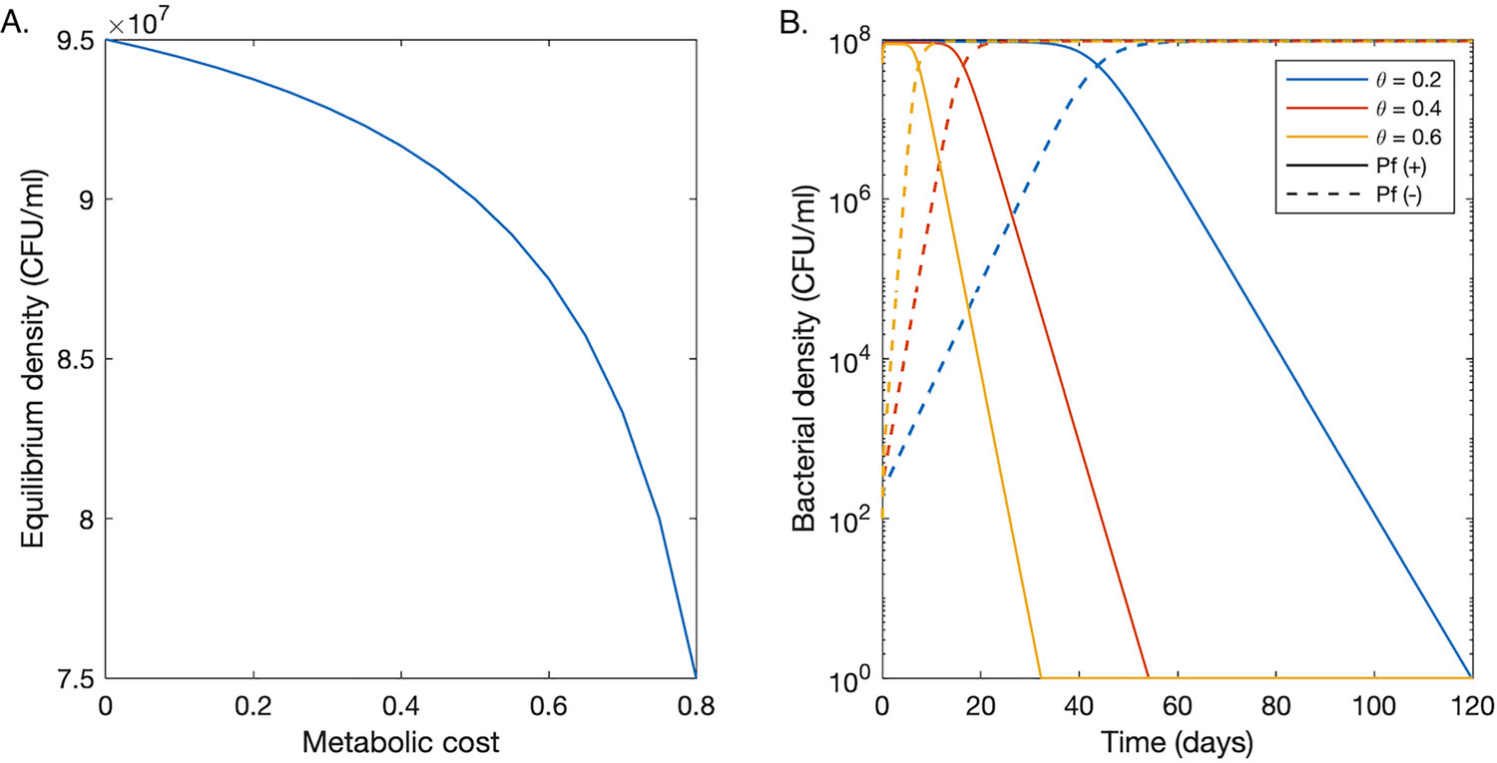
Growth dynamics of Pf− and Pf+ strains in the absence of antibiotics. (A) Bacterial density at equilibrium versus metabolic cost. A metabolic cost of zero corresponds to a Pf− strain. Other nonzero values are only relevant for a Pf+ strain. (B) A plot of the predicted bacterial densities of Pf+ (solid curves) and Pf− (dashed curves) bacteria versus time following the introduction of 100 Pf− bacteria into a Pf+ population at equilibrium. We consider three different metabolic costs for Pf+ strains.

The metabolic cost of phage production θ leads to a reduction in Pf+ strains per-capita growth rate and, ultimately, in a lower long-term equilibrium for Pf+ strains relative to Pf− strains (Fig. 2A). When in isolation (indirect competition), both strains grow to densities above 10^7^ CFU/ml, consistent with the number of genomic copies observed in CF lungs (7, 26). Pf+ and Pf− strains eventually reach their respective carrying capacity in independent infection sites: the carrying capacity of Pf− strains is equal to $K\times \;(1\;–\;\delta_{\text{B}}/r_{\text{max}})$ and exceeds Pf+ strains’ carrying capacity—equal to *K* × (1 − δ*_B_*/[*r*_max_(1 − θ)]—due to the extra energetic cost θ of phage production for Pf+ strains.

If a small population (10^2^ CFU/ml) of Pf− bacteria is introduced and directly competes with an established Pf+ bacterial colony, assuming θ> 0, the density of the Pf− strain increases over the course of multiple days until it reaches a density close to that of the Pf+ strain (Fig. 2B). The density of the Pf+ strain then decreases before eventually dropping below the extinction threshold of 1 CFU/ml. The time required for Pf− strains to outcompete Pf+ bacteria decreases with increasing metabolic cost of phage production. For a metabolic cost of 0.6 (corresponding to a 60% reduction in growth rate), it takes 10 days for the Pf− strain to exceed the Pf+ strain’s density and 32 days to drive the Pf+ strain extinct. Time to quasiextinction increases to 54 and 119 days for a metabolic cost of 0.4 and 0.2, respectively.

### Antibiotic treatment under direct competition between Pf+ and Pf− strains.

Pf+ strains cannot outcompete Pf− strains if both benefit from the sequestration of antibiotics by phages.

When competing directly for space and resources, Pf+ and Pf− bacterial strains experience the same phage and antibiotic concentrations in their environment. In this section, we assess the antibiotic effect on both strains living in direct competition, and we explore two antibiotic scenarios, namely: when the antibiotic effect is not dependent on the replication rate of the target bacteria (ε*_r_* = 0, equation 7), and when it is dependent on replication rate (ε*_r_* = 1).

**FIG 3:**
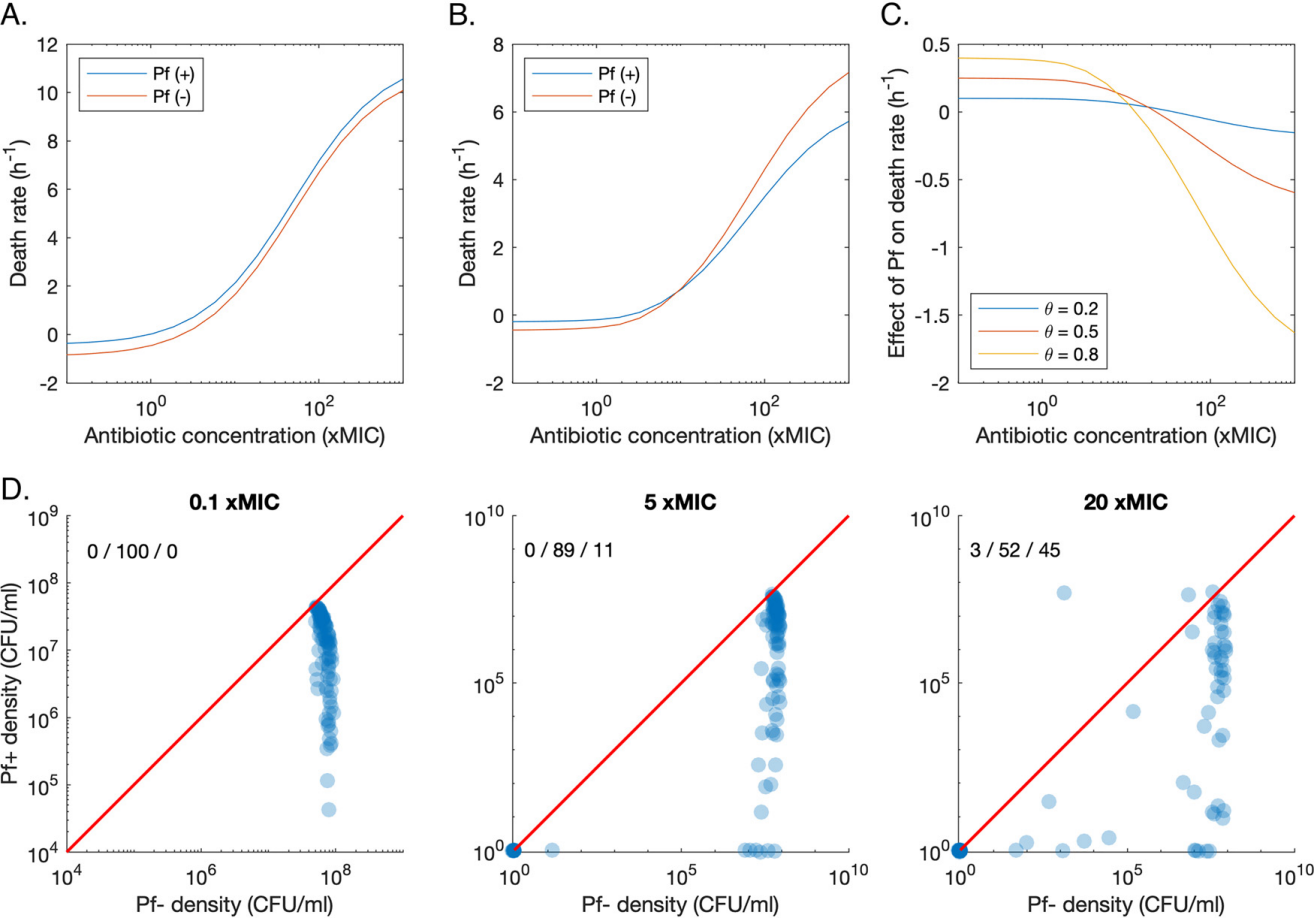
Growth dynamics of Pf− and Pf+ strains in direct competition during antibiotic treatment. (A) Plot of the net death rate of Pf+ and Pf− bacteria when the killing rate of the antibiotic is not dependent on the replication rate. (B) Plot of the net death rate of Pf+ and Pf− bacteria when the killing rate of the antibiotic is dependent on the replication rate. (C) Effect of phage production on the net death rate for different costs of phage production (θ). This effect is the difference between the growth of Pf+ and Pf− strains. (D) Pf+ density over Pf− density for three different concentrations of antibiotics (0.1×, 5×, and 20× MIC), administered twice a day, for 5 days. Each point corresponds to the median concentration over the last day of treatment, for a random parameter set, which was sampled from a uniform distribution in log space bounded by the parameter ranges in Table 1. The 1:1 line is given by the red line. The percentage of runs resulting in higher Pf+ density or Pf− density or the extinction of both strains is shown in the top left corner, in that order.

When the antibiotic effect is not dependent on replication rate (ε*_r_* = 0), the population growth rate of Pf− strains remains higher than the growth rate of Pf+ strains across the range of antibiotic concentrations (Fig. 3A). In this scenario, the Pf+ strain bears the energetic cost of phage production, while both strains benefit from the lower effective antibiotic concentrations due to phage sequestration of antibiotic molecules. As a consequence, the Pf− strain outcompetes the Pf+ strain at any antibiotic concentration that does not extirpate both strains.

We also investigated whether competitive exclusion of Pf+ strains coexisting with Pf− strains in the same infection sites can be prevented when antibiotics target specifically bacterial replication pathways (ε*_r_* = 1). In such a case, the benefit of phages’ protective biofilm is still equally shared between Pf+ and Pf− strains. However, the energetic cost θ of phage production (equation 2), sustained by Pf+ strains only, might be partially compensated by the strain-specific reduced sensitivity to antibiotics due to Pf+ lower reproductive rate (equation 7). Simulations showed that up to 10× MIC, the Pf− strain continues to have a lower death rate than the Pf+ strain. However, Pf+’s lower replication rate leads to a relative advantage compared to Pf− strains at higher antibiotic concentrations (Fig. 3B). The difference in death rate between Pf− and Pf+ bacteria at these elevated antibiotic concentrations increases as the metabolic cost of phage production, θ, increases (Fig. 3C). The reduced reproductive rate of Pf+ bacteria caused by phage production thus provides an additional mechanism for tolerance against antibiotics with replication-related targets. However, this additional benefit is usually insufficient to compensate for the reduction in replication rate and, ultimately, to reverse the outcome of competition between Pf+ and Pf− strains before both strains go extinct. For 100 random parameter sets (sampled within the ranges given in Table 1), none result in a higher density of Pf+ than of Pf− over the last day of antibiotic treatment, for antibiotic doses of 0.1× and 5× MIC (Fig. 3D). At an antibiotic dose of 20× MIC, 3 out of 100 parameter sets result in Pf+ having a higher density than Pf−. No parameter sets result in the extinction of Pf−, but not of Pf+. Therefore, in our model, Pf− outcompetes Pf+ under direct competition in more than 90% of the cases even when replication-targeting antibiotics are used.

**TABLE 1:** Model parameters

Symbol	Meaning	Value(s)^*a*^	Unit	Reference(s) or source
*H*	Hill coefficient	0.6−2.5 (0.8)		69, 71, 72
*r*_max_	Maximum growth rate	0.5−1.5 (1)	hour^−1^	73
θ	Metabolic cost of phage production	0.05−0.5 (0.2)		74, 75
*K*	Carrying capacity	10^8^	CFU/milliliter	76
Γ	Maximum killing rate	2−15 (12)	hour^−1^	69, 71, 77
ε*_r_*	Growth rate dependence of kill rate	0.1−0.9 (0.5)		78, 79
γ*_r_*	Growth rate for kill rate at half of its maximum	0.05−0.5 (0.2)	hour^−1^	78
ϕ	Antibiotic sequestration factor	10^5^−10^7^ (10^6^)	particle *V*^−1^	Text S1
*w*	Antibiotic mol wt	(468 × 10^6^)	microgram/mole	
*K_d_*	Binding dissociation factor	10^13^−10^15^ (10^14^)	particle	Text S1
ε*_k_*	Density dependence of *A*_50_	0.1−1 (1)		80
*k*_0_	Antibiotic concn for kill rate at half of its max (*A*_50_)	(3.7)	microgram/milliliter	71
ξ	Density-dependent maximum increase in *A*_50_	2−20 (10)	microgram/milliliter	80
γ*_k_*	Density for *A*_50_ at half of its maximum	10^6^−10^7^ (5 × 10^6^)	CFU/milliliter	80
δ*_B_*	Basal mortality rate	0.025−0.25 (0.05)	hour^−1^	
*A*_max_	Peak antibiotic concn in sputum	0.01−100	microgram/milliliter	81
δ*_a_*	Antibiotic decay rate	0.05−0.5 (0.1)	hour^−1^	44
λ	Phage production rate	0.1−10 (1)	hour^−1^	38
δ*_V_*	Phage decay rate	0.01−1 (0.1)	hour^−1^	82

a We provide the range of possible values we consider in our sensitivity analysis and give the exact value we use for tobramycin and *Pa* in parenthesis. These are the values we use by default.

10.1128/mSystems.00193-21.4 TEXT S1 Parameter estimations for parameters not found in the literature. Download Text S1, DOCX file, 0.02 MB. Copyright © 2021 Pourtois et al. 2021 Pourtois et al. https://creativecommons.org/licenses/by/4.0/ This content is distributed under the terms of the Creative Commons Attribution 4.0 International license.

### Antibiotic treatment of nonoverlapping infection sites (indirect competition).

**(i) The Pf+ strain is more likely to outcompete the Pf− strain at intermediate antibiotic concentrations (20× MIC).** In the case of nonoverlapping bacterial strains (indirect competition), phages are absent in Pf− strain environments, and thus, there is no change in the effective antibiotic concentration experienced by Pf− strains. In Pf+ bacterial infection sites, in contrast, phages can sequester some amount of the antibiotics, essentially lowering the local concentration of antibiotics acting on Pf+ bacteria.

**FIG 4:**
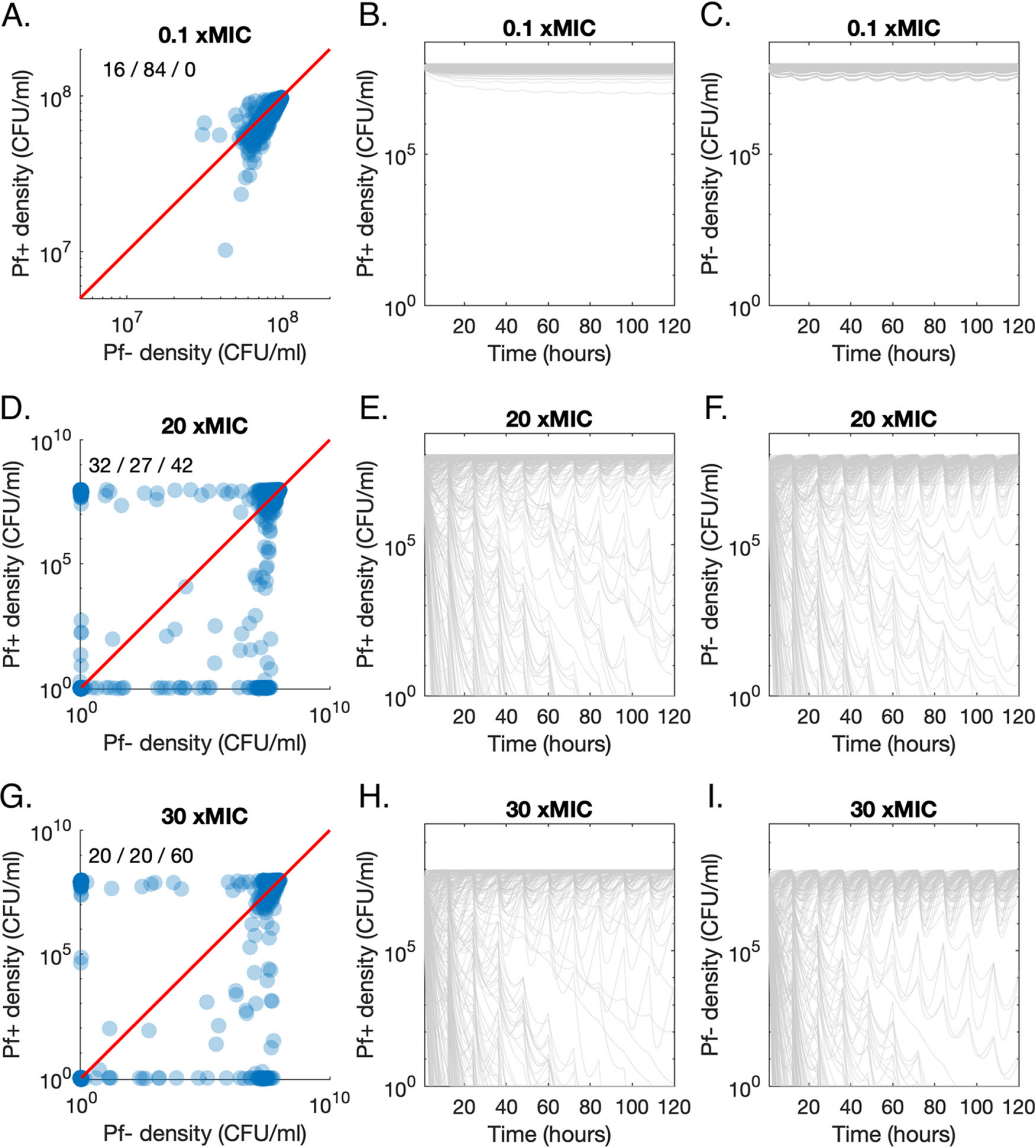
Growth dynamics of Pf− and Pf+ strains in direct competition during antibiotic treatment. (A) Pf+ density over Pf− density for 0.1× MIC administered twice a day, for 5 days. Each point corresponds to the median concentrations over the last day of treatment, for a random parameter set, sampled uniformly from ranges given in Table 1. The percentage of runs resulting in higher Pf+ density or Pf− density or the extinction of both strains is given in the top left corner, in that order. (B) Pf+ density over time for 0.1× MIC, administered twice a day, for 5 days. Each gray line corresponds to one random parameter set. (C) Associated Pf− density over time. Corresponding plots are shown in panels D, E, and F and G, H, and I for 20× MIC and 30× MIC, respectively.

At low antibiotic concentrations (doses of 0.1× MIC), the Pf− strain maintains a higher density than the Pf+ strain over the last day of treatment, for 84% of parameter sets (Fig. 4A). Both strains maintain a high density over 5 days (Fig. 4B and C). For doses of 20× MIC per day, the Pf+ strain outcompetes the Pf− strain for 32% of parameter sets and is outcompeted for 27% of parameter sets (Fig. 4D). This difference in proportion was significant (Χ^2^ = 5.11; *P* = 0.024; confidence interval [CI], 0.047 ± 0.041). Both Pf+ and Pf− strains go extinct in the remaining 42% of runs. At this intermediate antibiotic concentration, there is more variation between runs, with some parameter sets leading to rapid extinction, while others lead to slow declines over multiple days or oscillate under the antibiotic-free density (Fig. 4E and F). Finally, the proportion of runs that result in the extinction of both strains increases to 60% when the antibiotic concentration increases to 30 xMIC (Fig. 4G to I). At this concentration, Pf+ strains prevail as frequently as Pf− strains (20% each; Χ^2^ = 0.078, *P* = 0.78, CI, −0.042 ± 0.030).

**(ii) The fitness of the Pf+ strain relative to the Pf− strain is determined by different parameters at different antibiotic concentrations.** We performed a partial rank coefficient analysis to determine which parameters had the strongest relationships with Pf+ density, Pf− density, and the ratio of Pf+ to Pf− (see Fig. S1 and Table S1 in the supplemental material). When including antibiotic concentration as a variable, its effect dominated that of the other parameters. We thus calculated correlation coefficients for three different antibiotic concentrations per dose: 0.1× MIC, 5× MIC, and 20× MIC.

10.1128/mSystems.00193-21.1 FIG S1 Partial correlation coefficients between parameters and Pf+ density, Pf− density, and Pf+ to Pf− density ratio, with the antibiotic concentration per dose as a variable parameter (A) or set to 0.1× MIC (B), 5× MIC (C), or 20× MIC (D). Download FIG S1, TIF file, 0.5 MB. Copyright © 2021 Pourtois et al. 2021 Pourtois et al. https://creativecommons.org/licenses/by/4.0/ This content is distributed under the terms of the Creative Commons Attribution 4.0 International license.

10.1128/mSystems.00193-21.2 TABLE S1 *P* values for the hypothesis that there is no partial correlation between each parameter and Pf+ density, Pf− density, and the ratio between Pf+ and Pf− density, falling under four different possible ranges: below 0.01 (***), between 0.01 and 0.05 (**), between 0.05 and 0.1 (*) and above 0.1 (-). Download Table S1, DOCX file, 0.02 MB. Copyright © 2021 Pourtois et al. 2021 Pourtois et al. https://creativecommons.org/licenses/by/4.0/ This content is distributed under the terms of the Creative Commons Attribution 4.0 International license.

At the low antibiotic concentration of 0.1× MIC, the metabolic cost of phage production θ and bacterial death rates δ*_B_* were negatively correlated with the ratio of Pf+ to Pf− density (Fig. S1 and Table S1). The two strongest positive correlations were smaller in magnitude but still significant and were observed with the maximum growth rate and the maximum antibiotic killing rate. Together, this points to low net growth rate and low antibiotic effectiveness making a Pf+ strain uncompetitive compared to a Pf− strain. Phage-related parameters did not have significant effects at this concentration.

**FIG 5:**
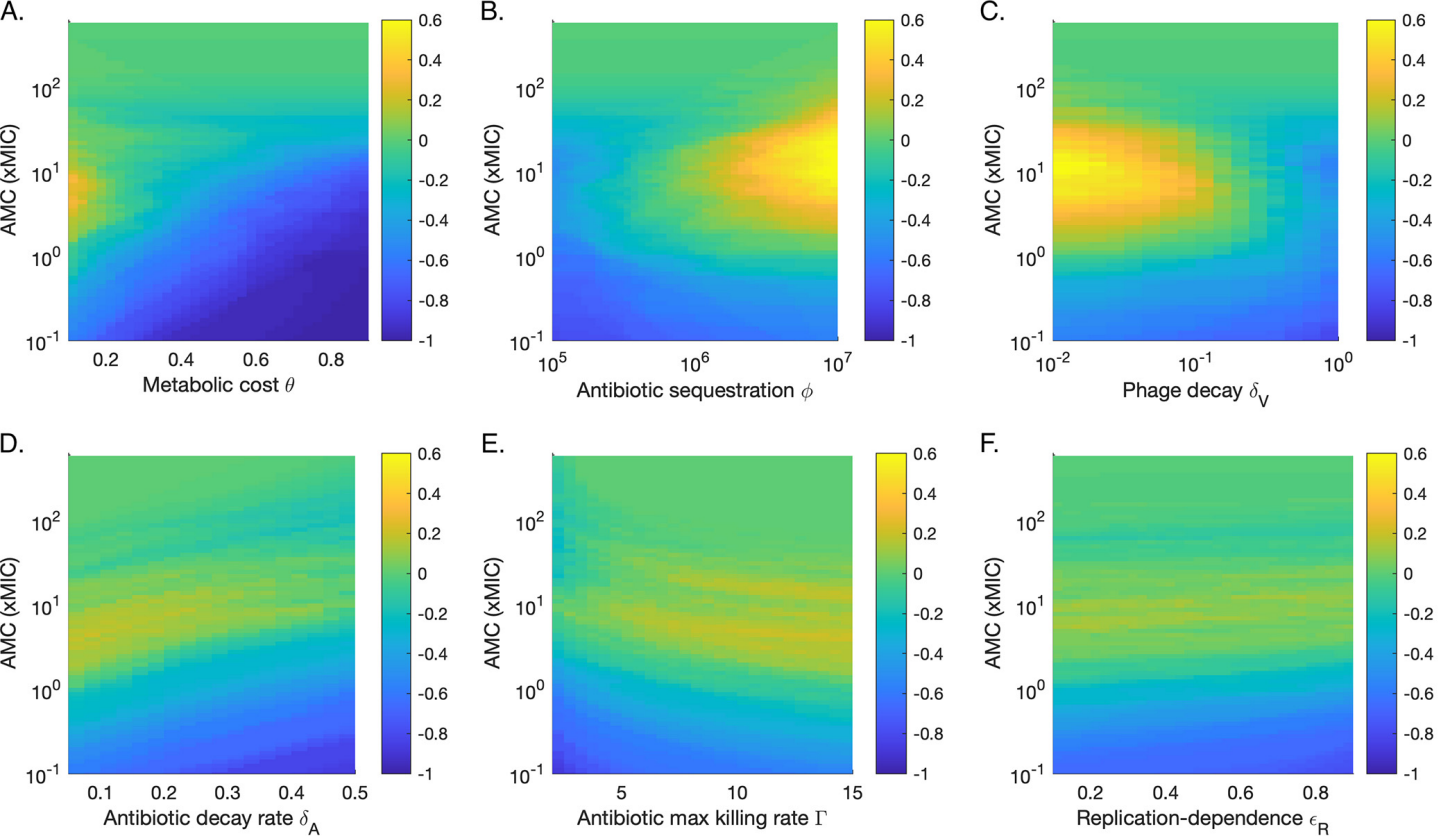
Sensitivity analysis for six key parameters (see Fig. S1 in the supplemental material). Color represents the difference between the proportion of runs for which the density of the Pf+ strain exceeds that of the Pf− strain over the last day and the proportion of runs for which the opposite occurs. For example, a value of 0 indicates that as many runs were dominated by Pf+ as by Pf− strains. The density used for each run is the median density over the last day of antibiotic treatment. (A) Metabolic cost. (B) Antibiotic sequestration. (C) Phage decay rate. (D) Antibiotic decay rate. (E) Antibiotic maximum killing rate. (F) Replication dependence of antibiotics.

The metabolic cost continued to be highly negatively correlated with Pf+ relative fitness at concentrations of 5× and 20× MIC (Fig. S1). At these higher concentrations, a low metabolic cost allows for the fitness of the Pf+ strain to be higher than the fitness of the Pf− strain, on average (Fig. 5A and Table S2). Unlike at smaller concentrations, antibiotic sequestration by phages, and phage decay were significantly correlated with Pf+ competitiveness (Fig. S1, Table S1, and Table S2). High antibiotic sequestration and low phage decay lead to a high relative Pf+ fitness at concentrations of 5× MIC (Fig. 5B and C and Table S2).

10.1128/mSystems.00193-21.3 TABLE S2 Estimate and level of significance (***, below 0.01) for the intercept and slope of each linear model. Each model includes an intercept, a parameter, antibiotic concentration, a quadratic term for antibiotic concentration, and an interaction between the parameter and antibiotic concentration. Download Table S2, DOCX file, 0.01 MB. Copyright © 2021 Pourtois et al. 2021 Pourtois et al. https://creativecommons.org/licenses/by/4.0/ This content is distributed under the terms of the Creative Commons Attribution 4.0 International license.

In addition to metabolic cost and antibiotic sequestration, phage production and antibiotic decay were both positively correlated with Pf+ relative fitness when 5× MIC was used (Fig. S1 and Table S1). Up to 20% more runs are dominated by Pf+ strains than by Pf− strains, with the highest proportion found for low decay rates (Fig. 5D). The range of antibiotic concentrations that leads to the highest Pf+ relative fitness increases as the antibiotic decay rate increases (Fig. 5D and Table S2). Finally, the maximum killing rate of the antibiotic also has a significant positive impact on the ratio of Pf+ to Pf− density (Fig. S1). As antibiotic concentration increases, the killing rate leading to the highest average Pf+ fitness dominated runs decreases (Fig. 5E and Table S2). The highest advantage for Pf+ compared to Pf− occurs for antibiotics with high killing rates at intermediate concentrations (Fig. 5). Unlike for direct competition, increasing degrees of replication-dependent antibiotics have a small negative effect on the relative fitness of Pf+ (Fig. 5F and Table S2). However, replication dependence becomes more beneficial to Pf+ as antibiotic concentration increases (Table S2).

**FIG 6:**
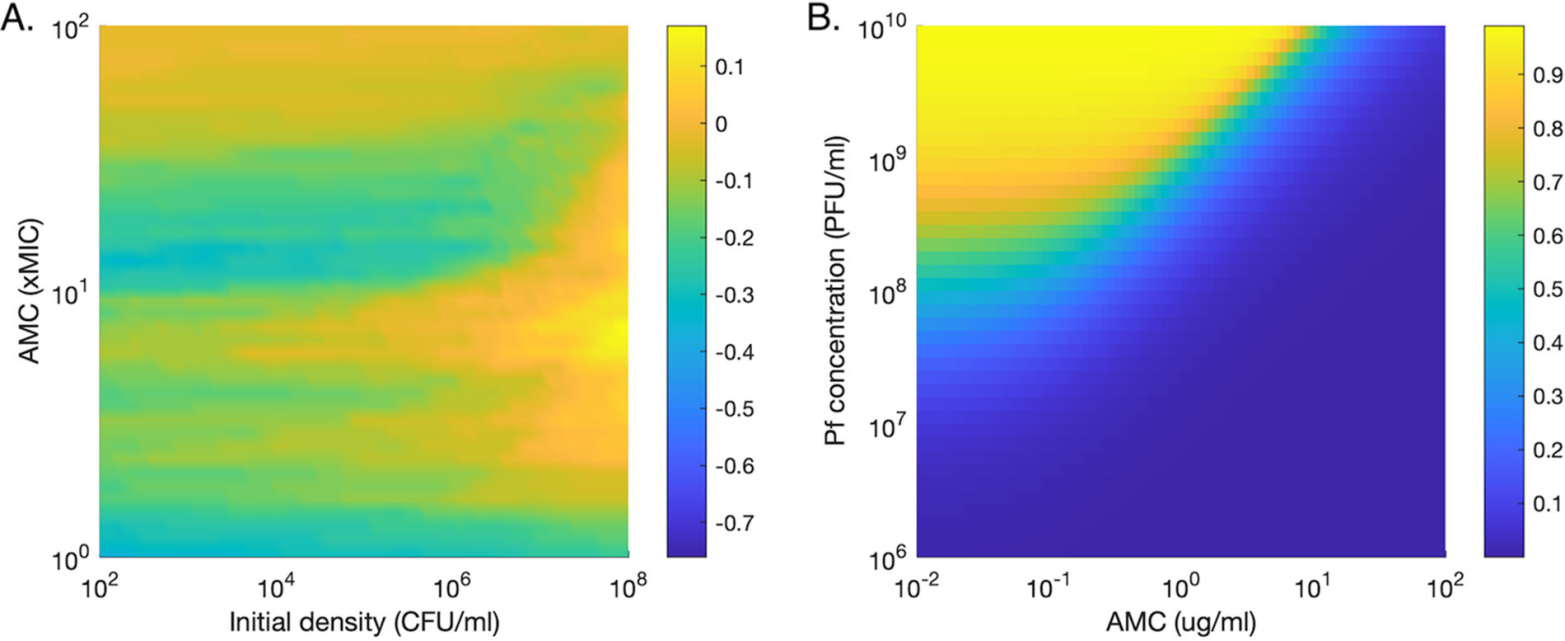
Effect of initial conditions on *Pa* survival and antibiotic sequestration. (A) Effect of the initial bacterial density. Difference between the proportion of runs for which the density of the Pf+ strain exceeds that of the Pf− strain, and the proportion of runs for which the opposite occurs. For example, a value of 0 indicates that as many runs were dominated by Pf+ strains as by Pf− strains. The density used for each run is the median density over the last day of antibiotic treatment. (B) Percentage of antibiotic sequestered over a range of starting antibiotic concentrations and phage concentration when ϕ is 10^6^.

**(iii) High initial bacterial density is necessary for phages to provide nonnegligible benefits.** Pf+ relative fitness slowly increases as the initial bacterial concentration (before the start of the antibiotic treatment) increases, with a sharp increase at densities close to the carrying capacity of 10^8^ CFU/ml (Fig. 6A). This increase is strongest for antibiotic concentrations around 10× MIC. A combination of high initial densities and intermediate antibiotic concentrations is necessary for initial phage concentrations to provide adequate protection to a Pf+ strain. In our model, phages at densities of 10^9^ PFU/ml (corresponding to bacterial densities of approximately 10^8^ CFU/ml) sequester 80% of antibiotics at a concentration of 1× MIC, but this measure drops sharply as phage density decreases (Fig. 6B).

## DISCUSSION

Although most bacteriophages have traditionally been thought to have only parasitic relationships with their bacterial hosts, the discovery of commensalistic and even mutualistic phage-bacterium relations suggests that a deeper understanding of phage biology is clinically important. In particular, filamentous phages have been found to affect the fitness of their hosts in a variety of ways, often increasing their virulence (43). With the discovery that filamentous phages of *Pa* lead to more resistant biofilms and protection against many antibiotics, we set out to use a mathematical approach to investigate how antibiotic sequestration by Pf phages impact the fitness of *Pa* in different antibiotic environments. Namely, we wanted to understand whether certain bacterial or antibiotic characteristics may be contributing to the emergence and resilience of Pf+ *Pa* infections. These findings could also prove informative for other bacteria such as Escherichia coli, Klebsiella pneumoniae, Neisseria meningitidis, Neisseria gonorrhoeae, and many *Vibrio* species, which are also infected by filamentous phages (30, 49–53), and are relevant to the clinical world.

We found that in the absence of antibiotics, Pf− bacterial strains outcompeted Pf+ strains, as expected given the energetic cost of phage production. This outcome is well-known in ecology as the principle of competitive exclusion for species exploiting the same resources, also known as Gause’s law (54). This is consistent with the low Pf phage prevalence in pediatric patients with CF, many of whom have received relatively limited antibiotic treatments (7). In direct competition, assuming that Pf− strains cannot be infected by phages, Pf+ strains are unable to outcompete Pf− strains in most simulations, because Pf+ strains sustain the energetic cost of phage production but share the benefits evenly with Pf− strains. A low percentage of parameter combinations resulted in a higher Pf+ than Pf− density when high concentrations of antibiotics with replication-related targets were used. Under these conditions, the metabolic cost associated with phage production provides some protection against antibiotics. However, it is unlikely that such a limited fraction of the parameter space could drive the increase in Pf+ strains in patients with CF.

Overall, our model suggests that Pf+ strains can consistently outcompete Pf− strains only when Pf+ strains do not overlap in the same infection sites with Pf− strains and thus, while bearing the cost of phage production, they do not share the benefit with Pf− strains. A recent report demonstrated that Pf+ bacteria grown *in vitro* may encase themselves within bundles of phage, thus providing a shielding mechanism against antibiotics (29). It is unclear, however, if this behavior occurs in clinical settings or if Pf+ bacteria can protect Pf− bacteria against antibiotics within a mixed-strain infection. It is conceivable that Pf+ strains may be able to outcompete Pf− strains due to this highly localized phage presence. Our model simplifies these questions by using strict definitions of direct and indirect competition. Further studies are necessary to understand these coculture biofilms, particularly in clinically relevant settings.

Interestingly, Pf+ strains are not able to dominate in all indirect competition scenarios. Intermediate concentrations of antibiotic (5× to 25× MIC) are critical for Pf+ strains to significantly outcompete Pf− strains. These concentrations correspond to the regimen at which antibiotic sequestration by phages lowers the effective antibiotic concentration—thus lowering the death rate of Pf+ bacteria—enough to compensate for the higher energetic cost of phage production. Under these circumstances, Pf+ bacterial density exceed that of Pf− bacteria. Below these antibiotic concentrations, the marginal benefit of a reduction in antibiotic effective concentration accrued thanks to the protective effect of the phage biofilm is unable to compensate for the cost of phage production, and thus Pf+ density remains lower than that of Pf− strain. At high antibiotic doses, the ability of phages to sequester antibiotic is overwhelmed, and infections with both Pf+ and Pf− are eventually cleared. This is consistent with the clinical evidence that shows Pf+ strain infections are associated with chronic infections (7) that have presumably been treated with inhaled antibiotics. Once a patient with CF has failed eradication, chronic inhaled antipseudomonal therapy is initiated with inhaled tobramycin, or alternatively aztreonam. While the sputum antibiotic levels achieved with inhaled antibiotics are reported as well above MIC (55, 56), it is plausible that some areas of infected lung tissue do not reach such high levels. CF is an obstructive pulmonary disease which is characterized by areas of heterogeneous ventilation (57, 58). There are likely distal areas of the lung with poor ventilation that do not see the same delivery of inhaled antibiotics (59), thus creating an environment with concentrations of antibiotics that favor the survival and dominance of Pf+ strains. The continuous use of antibiotics at sublethal concentrations may thus be partially responsible for driving infections toward a Pf+ dominated state. In particular, our model suggests that the use of antibiotics with high killing rates and low decay rates is more likely to lead to high fitness for Pf+ strains. If possible, the ideal treatment for avoiding Pf+ bacterial infections would use antibiotics that cannot be sequestered by phages, such as ciprofloxacin (7, 60). In addition, treating infections early would allow antibiotics to act before phage density is high enough to lead to significant sequestration of antibiotics. We focused here on antibiotics delivered via inhalation as part of medical treatment. However, antibiotic substances can also be secreted by bacteria to prevent interspecies competition (48, 61). Our results thus inform our understanding of bacterial competition in a wide range of contexts, as well as in the clinical setting on which we focused in this work.

In this model, we described the effects of antibiotic sequestration by Pf phages on biofilm-embedded bacteria under antibiotic stress. Our results suggest that antibiotic sequestration by Pf could drive an increase in Pf+ strains, under the regular use of antibiotics that is characteristic of treatment in CF. However, there are many additional factors that will need to be considered in order to understand the full effect of phages on bacterial communities in the lung. First, we have not accounted for how the presence of phages may change other properties of the infection site that affect *Pa* survival. For example, Pf phages may impact the rheological properties of the infection environment, the adhesivity of the biofilm, the transport of nutrients and antibiotics, as well as any immune response. In turn, these properties can affect the physiological state of bacteria, and phage production (62). In addition, following previous modeling efforts (38, 63), we assumed that bacterial growth can be described by a simple logistic model, a hypothesis that, although realistic, still needs to be validated through empirical studies. Also, we did not include the dynamics of colonization from different localized lung sections that could lead to more complicated source-sink dynamics of the strain types or competition-colonization trade-offs. Finally, we simulated bacterial dynamics over days, whereas changes in bacterial communities in the lung occur over multiple years. Long-term processes such as the evolution of antibiotic resistance and spatial movement within the lung are likely to influence the distribution of Pf+ strains and be relevant to treatment design for CF. For these reasons, the results of our modeling study should not be taken as prescriptive. Rather, the aim of our work was to explore, under plausible hypotheses and realistic values of model parameters, the competitive dynamics of Pf− and Pf+ strains under antibiotic treatment. As the scientific understanding of the additional factors and processes driving the dynamics of Pf− and Pf+ strains improves with new laboratory and clinical research, it will be possible to include them in quantitative models of bacterial infection under antibiotic treatment and increase their explanatory and predictive power.

### Conclusion.

Filamentous phages have increasingly been recognized as important players in the development of chronic infections through their effect on antibiotics, immunity, and biofilm formation. In particular, Pf+ bacterial infections in patients with CF are associated with chronic infection and a larger decrease in lung function during exacerbation. Our simulations showed that the fitness of Pf+ strains is highest when antibiotic sequestration by phages is high but localized and when a constant intermediate concentration of antibiotics is maintained in the lungs. This suggests that the frequent antibiotic treatments often used in CF care could contribute to the increased prevalence of Pf+ strains in patients with CF. Our model suggests that high sequestration, high antibiotic killing rate, and low antibiotic decay rates particularly favor Pf+ strains. Of note, phages have additional effects on bacteria and the human immune system that are still poorly understood and could not be captured in our model. A better understanding of the ecology of phages in the human body will be an essential step for developing more successful treatment strategies for bacterial infections.

## MATERIALS AND METHODS

### General framework.

We envisioned two alternative (i.e., mutually exclusive) types of interaction between Pf+ and Pf− strains: (i) direct competition in a mixed population of Pf− and Pf+ bacterial strains coexisting in the same location (e.g., in direct contact and within the same portion of the lung airway) and (ii) no direct interaction with Pf− and Pf+ strains coexisting in the same patient but established in different (i.e., nonoverlapping) locations, e.g., spatially separated infection sites in the lungs (Fig. 1A)—hereafter referred to also as indirect competition. While Pf− and Pf+ strains do not interact directly, their relative growth rates and abundances affect their probability of transmission to noninfected areas of the lungs.

In direct competition, both Pf− and Pf+ strains must compete for the same nutrients and space. As filamentous phages are generally highly specific (64, 65), we assumed that the Pf− strain cannot be infected by phages produced by the Pf+ strain. However, under antibiotic treatment, the Pf− strain can benefit from the protective biofilm of filamentous phages produced by the Pf+ strain coexisting in the same location.

In indirect competition, there is no interstrain competition for nutrients and space, and we assumed that, under antibiotic treatment, the Pf− strain does not benefit from the protective effect of phages produced elsewhere by the Pf+ strain.

### Model description.

**(i) Bacterial growth.** In the absence of antibiotics, bacteria *B_i_* (in CFU/milliliter) from each strain *i* = Pf+ or Pf−, replicate at a density-dependent rate Φ(*B*_tot_) and die at the per-capita rate δ*_B_*:

(1) $$\frac{dB_{i}}{\mathrm{dt}}=\Phi \left(B_{\text{tot}}\right)B_{i}\;-\;\delta_{B}B_{i}$$

We choose to model bacterial growth directly with a logistic equation rather than a Gompertz equation or a mechanistic approach with nutrients, as it only requires estimating the maximum growth rate and carrying capacity. This model and its variants are used regularly to describe bacterial growth (38, 63, 66, 67). We thus model the per-capita replication rate Φ(*B*_tot_) as a decreasing logistic function of the total bacterial concentration in the local biofilm *B*_tot_:

(2) $$\Phi \left(B_{\text{tot}}\right)=r_{\text{max}}\left(1\;-\;\frac{B_{\text{tot}}}{K}\right)\left(1\;-\;\theta \right)$$

where *r*_max_ is the absolute maximum replication rate, *K* is the bacterial density at which the per-capita reproductive rate Φ(*B*_tot_) is equal to zero, and θ is the reduction in the per‐capita replication rate caused by the metabolic cost of phage production in Pf+ bacterial strains only (θ is equal to zero for Pf− strains). When both strains are competing directly for space and resources, the total bacterial concentration *B*_tot_ is the sum of their concentrations, namely: *B*_tot_ = *B*^+^ + *B*^−^. When competing indirectly, *B*_tot_ is set equal to the local concentration of bacteria, i.e., either the concentration of the Pf+ strain or the Pf− strain.

**(ii) Phage production.** Filamentous phages *V* (PFU/milliliter) are produced by Pf+ bacteria at a constant rate λ and decay at a rate δ*_V_*:

(3) $$\frac{\mathrm{dV}}{\mathrm{dt}}\;=\lambda B^{+}\;-\;\delta_{V}V$$

Both λ and δ*_V_* were numerically optimized (see “Parametrization”) to obtain viral densities an order of magnitude larger than bacterial densities, consistent with literature reports of *Pa* infections (7).

**(iii) Antibiotic treatment.** We consider a range of antibiotic regimens, with different antibiotic concentrations per dose, administered twice a day. We assume each dose of antibiotics leads to an instantaneous peak *A*_max_ in the sputum. All concentrations discussed in this work represent concentrations in the sputum, which can differ in nontrivial ways from the original concentration administered orally. After administration, antibiotics are removed from the system via both degradation of the drug and clearance due to natural flow through the body. Here, we assumed that their concentration follows first order kinetics (68), i.e., it decays at a constant rate δ*_A_* until the next treatment:

(4) $$\frac{\mathrm{dA}}{\mathrm{dt}}\;=\;-\delta_{A}A$$

We thus impose a cyclic pattern to antibiotic concentrations, and as a result, to bacterial density and phages as well (Fig. 1B to D).

To describe the effects of antibiotics on the bacterial infection, we built upon, and extended, the original model developed by Levin and Udekwu (44). Specifically, the effect of antibiotics in the system is accounted for by modifying equation 1 as follows:

(5) $$\frac{dB_{i}}{\mathrm{dt}}\;=\Phi \left(B_{\text{tot}}\right)B_{i}\;-\;\delta_{B}\;B_{i}\;-\;\Psi \left(A,B,V\right)B_{i}\;$$

where Ψ(*A*, *B*, *V*) is the per-capita reduction in bacterial growth rate or, equivalently, the increase in mortality rate as a consequence of antibiotics at concentration *A*, bacterial density *B*, and phage density *V*—hereafter referred to as the antibiotic killing rate. Ψ(*A*, *B*, *V*) is here described by a Hill function (69), i.e., an increasing function of the effective antibiotic concentration *A*_eff_(*A*, *V*) that levels off to $\psi \left(\Phi \left(B_{\text{tot}}\right)\right)$, the maximum killing rate due to antibiotics:

(6) $$\Psi \left(A,B,V\right)=\;\frac{\psi \left(\Phi \left(B_{\text{tot}}\right)\right)}{1\;+\;{\left(\frac{A_{50}(B)}{A_{\text{eff}}(A,V)}\right)}^{H}}\;$$

where *A*_50_(*B*) is the antibiotic concentration leading to half $\psi \left(\Phi \left(B_{\text{tot}}\right)\right)$, and *H*, the Hill parameter, is proportional to the steepness of the function at *A*_eff_ = *A*_50_ (44).

The effective maximum killing rate $\;\psi \left(\Phi \left(B_{\text{tot}}\right)\right)$ of the antibiotic is a function of the absolute maximum killing rate Γ and the effect of bacterial replication rate on the antibiotic efficacy (44):

(7) $$\psi \left(\Phi \left(B_{\text{tot}}\right)\right)=\Gamma \left(1\;-\;\varepsilon_{r}\right)\;+\;\varepsilon_{r}\Gamma \left(\frac{\Phi \left(B_{\text{tot}}\right)}{\Phi \left(B_{\text{tot}}\right)\;+\;\gamma_{r}}\right)$$

The parameter ε*_r_*, bounded between 0 and 1, describes the degree to which a particular antibiotic’s efficacy depends on the growth rate, i.e., it does not depend upon bacterial reproductive rate $\Phi (B_{\text{tot}})$ when ε*_r_* = 0, it depends entirely upon bacterial reproductive rate when ε*_r_* = 1. The parameter γ*_r_* represents the growth rate at which the rate of killing is half of its maximum when ε*_r_* = 1.

The effective antibiotic concentration is determined by the concentration of antibiotics that are not sequestered by phages. Following Hulme and Trevethick (70), to describe the extent of sequestration, we use the equilibrium expression for ligand-receptor binding as binding dynamics occur at a much faster scale than bacterial killing:

(8) $$A_{\text{eff}}(A,V)=A\;-\;\frac{w\left(\left(A_{m}\;+\;\phi V\;+\;K_{d}\right)\;-\;\sqrt{{\left(A_{m}\;+\;\phi V\;+\;K_{d}\right)}^{2}\;-\;4A_{m}\phi V}\right)}{2N_{A}}$$

where ϕ represents the number of antibiotic binding sites per phage (also referred to as antibiotic sequestration factor), $K_{d}$ the equilibrium dissociation constant, and $A_{m}$ the antibiotic concentration in molecules per milliliter. *w* and *N_A_* stand for the molecular weight of the antibiotic and Avogadro’s number, respectively, and are used for the conversion from microgram per milliliter to molecule per milliliter:

(9) $$A_{m}=\frac{AN_{A}}{w}$$

The effective *A*_50_—the antibiotic concentration leading to half of the maximum killing rate—is here assumed to be an increasing and saturating function of bacterial density *B*_tot_ (44), namely:

(10) $$A_{50}(B)=k_{0}\;+\;\varepsilon_{k}\xi \left(\frac{B_{\text{tot}}}{B_{\text{tot}}\;+\;\gamma_{k}}\right)$$

Accordingly, *A*_50_ ranges between *k*_0_ at low bacterial densities and *k*_0_ + ξ at high bacterial density, where ξ is the maximum additional antibiotic concentration that can be tolerated at high densities, γ*_k_* is the bacterial density at which *A*_50_ increases by half of its maximum amount and $\varepsilon_{k}$ is a switch parameter set to 0 if *A*_50_ is assumed to be independent from bacterial density and to 1 otherwise.

### Parametrization.

The equations in this model are general and can be applied to a variety of environments, bacteria, and antibiotics. Here, we parametrized our model to represent the growing conditions of *Pa* in CF lungs under tobramycin treatment, an antibiotic often used to treat *Pa* infections (Table 1). We chose a value in the middle of the range observed in the literature for most parameters relating to bacterial growth and phage production. We used parameter values from studies of *Pa* whenever available, but the ranges given in Table 1 apply to many bacterial species. For parameters describing the action of antibiotics, we give the range observed across a wide variety of antibiotics, as well as the particular value we used for tobramycin. Finally, we used numerical simulations to estimate the values of parameters whose value is not found in the literature (see Text S1 in the supplemental material). We performed a global sensitivity analysis to explore how bacterial density responds to variation in parameters around the fixed values used in our model (see Fig. S1 and Table S1 in the supplemental material). In this work, we were concerned with the effect of phage production on fitness, and we considered in our analysis a wide range of values for relevant parameters (metabolic cost and antibiotic sequestration constant). Time is measured in hours.

### Analysis.

All the simulations were performed using MATLAB. Growth rates were obtained directly from the equations above. We calculated the MIC by setting equation 5 to zero and solving for the antibiotic concentration, for parameters shown in parentheses in Table 1. Because of the deterministic nature of this model, this value represents the antibiotic concentration at which no growth occurs in all populations represented by those parameters. The MIC used as the unit for the figures is calculated from this single set of parameters (shown in Table 1) and kept fixed at the resulting value of 0.17 μg/ml for consistency. For example, changes in the metabolic cost from phage production would lead to changes in MIC, but the fixed value of 0.17 (corresponding to a metabolic cost of 0) is used to scale all graph axes.

For simulations, we used the differential equation solver *ode45* to compute the bacterial density of Pf+ and Pf− strains over a minimum of 5 days, for various antibiotic concentrations per dose, administered twice a day. We then used the log ratio of the median bacterial density for each strain in the last day of each simulation as a metric for comparison across different antibiotic regimens. We assumed that infection was cleared when the bacterium’s density dropped below the quasiextinction threshold of 1 CFU/ml.

To assess the sensitivity of the outcome to model parameters, we performed a partial rank correlation coefficient analysis (PRCC) with Latin hypercube sampling (LHS) after log transformation of model parameters over the ranges reported in Table 1. We generated 100 random parameter sets for the results presented in Fig. 3, 5, and 6 and 1,000 random parameter sets for Fig. 4 and the partial rank correlation coefficient analysis. For each of these parameter sets, we ran a simulation according to the description above. We refer to each of these simulations as a “run” in the Results. A proportion test was then used to compare the proportion of runs dominated by Pf+ and Pf− bacteria for different antibiotic concentrations. We further analyzed the effects of the six parameters (θ, ϕ, δ*_v_*, δ*_A_*, Γ, and ε*_r_*) and bacterial initial density on Pf+ relative fitness. We calculated the difference between the proportion of runs dominated by Pf+ bacteria and the proportion dominated by Pf− bacteria for a range of each parameter and antibiotic concentration. We then fit a linear model with an interaction term between parameter and antibiotic concentration and a quadratic term for antibiotic concentration. Results are reported in Table S2. We used R to perform the proportion test and model fitting.

### Data availability.

All code is available on GitHub at https://github.com/jpourtois/phage-antibiotics.

## Supplementary Material

Reviewer comments
