# Supplementary material for: Filamentous Bacteriophages and the Competitive Interaction between Pseudomonas aeruginosa Strains under Antibiotic Treatment: a Modeling Study
Source: mSystems. 2021 Jun 22;6(3):e00193-21. doi: 10.1128/mSystems.00193-21 (PMC8269214; doi:10.1128/mSystems.00193-21)
Supplement: TEXT S1 [file msystems.00193-21-s0001.docx]

**Supplementary Text S1**

**Parameter estimation**

All parameter values were obtained from the literature except for the metabolic cost of phage production $\theta$, the antibiotic sequestration factor $\phi$ and the binding dissociation factor $K_{d}.$ Precise values are not yet available in the literature for these parameters. Here we discuss how we chose the ranges considered in this model.

Metabolic cost $\theta$

Previous modeling work includes metabolic costs up to 50% (1). In addition, *P. aeruginosa* exhibits a moderate reduction in growth and bacterial density when infected by Pf phages (2). We set as the default a 20% reduction in growth rate when producing phages. We also provided death rates for $\theta=0.5$ and $\theta=0.8$(Fig. 3C ). Finally, we considered a wider range of values (0.1-0.9) in a sensitivity analysis (Figure 5A).

Antibiotic sequestration constant $\phi$

We define the antibiotic sequestration constant as the number of antibiotic molecules that can bind to one phage. We estimated a plausible range using numerical simulations, and verified that this range is compatible with the physical properties of both phage and antibiotics, as well as exisiting experiments. Sequestration constants at or below 10^5^ molecules per phage did not lead to net increase in density in our model (Fig. 5B). Values at or above 10^7^ molecules per phage led to large reductions in death rates over a wide range of antibiotic concentrations. As a conservative default estimate of $\phi$ for all simulations, we used 10^6^ molecules per phage.

To obtain a first estimate of the surface area available for binding, we consider Pf phages as simple cylinders, with a radius *r* of 3.5nm and a length *h* of 2000nm (3). We can then approximate the surface area *S_A_* of one phage as:

$$S_{A}=2\pi rh+\pi r^{2}=4.4 {10}^{4} nm^{2}$$

In order for a phage to sequester 10^6^ antibiotic molecules, it would be necessary to pack slightly more than 20 molecules per nm^2^. In the presence of negatively-charged surfaces , aminoglycosides such as tobramycin aggregate to form long and thin (7 x 1 nm) fibers (4), which could allow for tight packing around phages.

While the number of antibiotic molecules sequestered per phage has not been calculated directly, Tarafder et al. found that phage concentrations of 5 x 10^6^ pfu/ml and higher were effective at protecting *Pa* from antibiotics (5). This is much less than the phage concentration required when the sequestration constant is 10^6^, suggesting that our estimate is actually conservative. Further laboratory research will be necessary to confirm these estimates.

Binding dissociation factor$K_{d}$

Binding dissociation factors describe the ratio of the rate of dissociation over the rate of association for a ligand-receptor system. To our knowledge, binding dynamics between phages and antibiotics have not been quantitively investigated yet. Below 10^13^ molecules/ml, the effect of K_d_ becomes negligible (i.e. phages sequester the maximum amount of antibiotics). Above 10^15^ molecules/ml, the proportion of antibiotics bound to phages approaches 0. We use the intermediate value of 10^14^ as the default value in our model and consider values within this range in a sensitivity analysis (Figure S1).

**References**

1. Clifton SM, Kim T, Chandrashekhar JH, O’Toole GA, Rapti Z, Whitaker RJ. 2019. Lying in Wait : Modeling the Control of Bacterial Infections via Antibiotic-Induced Proviruses. mSystems 4:1–16.

2. Secor PR, Michaels LA, Smigiel KS, Rohani MG, Jennings LK, Hisert KB, Arrigoni A, Braun KR, Birkland TP, Lai Y, Hallstrand TS, Bollyky PL, Singh PK, Parks WC. 2017. Filamentous Bacteriophage Produced by Pseudomonas aeruginosa Alters the Inflammatory Response and Promotes Noninvasive Infection In Vivo. Infect Immun 85:1–11.

3. Secor PR, Burgener EB, Kinnersley M, Jennings LK, Roman-Cruz V, Popescu M, Van Belleghem JD, Haddock N, Copeland C, Michaels LA, de Vries CR, Chen Q, Pourtois J, Wheeler TJ, Milla CE, Bollyky PL. 2020. Pf Bacteriophage and Their Impact on Pseudomonas Virulence, Mammalian Immunity, and Chronic Infections. Front Immunol. Frontiers Media S.A.

4. Kopaczynska M, Lauer M, Schulz A, Wang T, Schaefer A. 2004. Aminoglycoside Antibiotics Aggregate To Form Starch-like Fibers on Negatively Charged Surfaces and on Phage λ -DNA. Langmuir 20:9270–9275.

5. Tarafder AK, von Kügelgen A, Mellul AJ, Schulze U, Aarts DGAL, Bharat TAM. 2020. Phage liquid crystalline droplets form occlusive sheaths that encapsulate and protect infectious rod-shaped bacteria. Proc Natl Acad Sci 201917726.
