# Supplementary figures and images for: Filamentous Bacteriophages and the Competitive Interaction between Pseudomonas aeruginosa Strains under Antibiotic Treatment: a Modeling Study
Source: mSystems. 2021 Jun 22;6(3):e00193-21. doi: 10.1128/mSystems.00193-21 (PMC8269214; doi:10.1128/mSystems.00193-21)

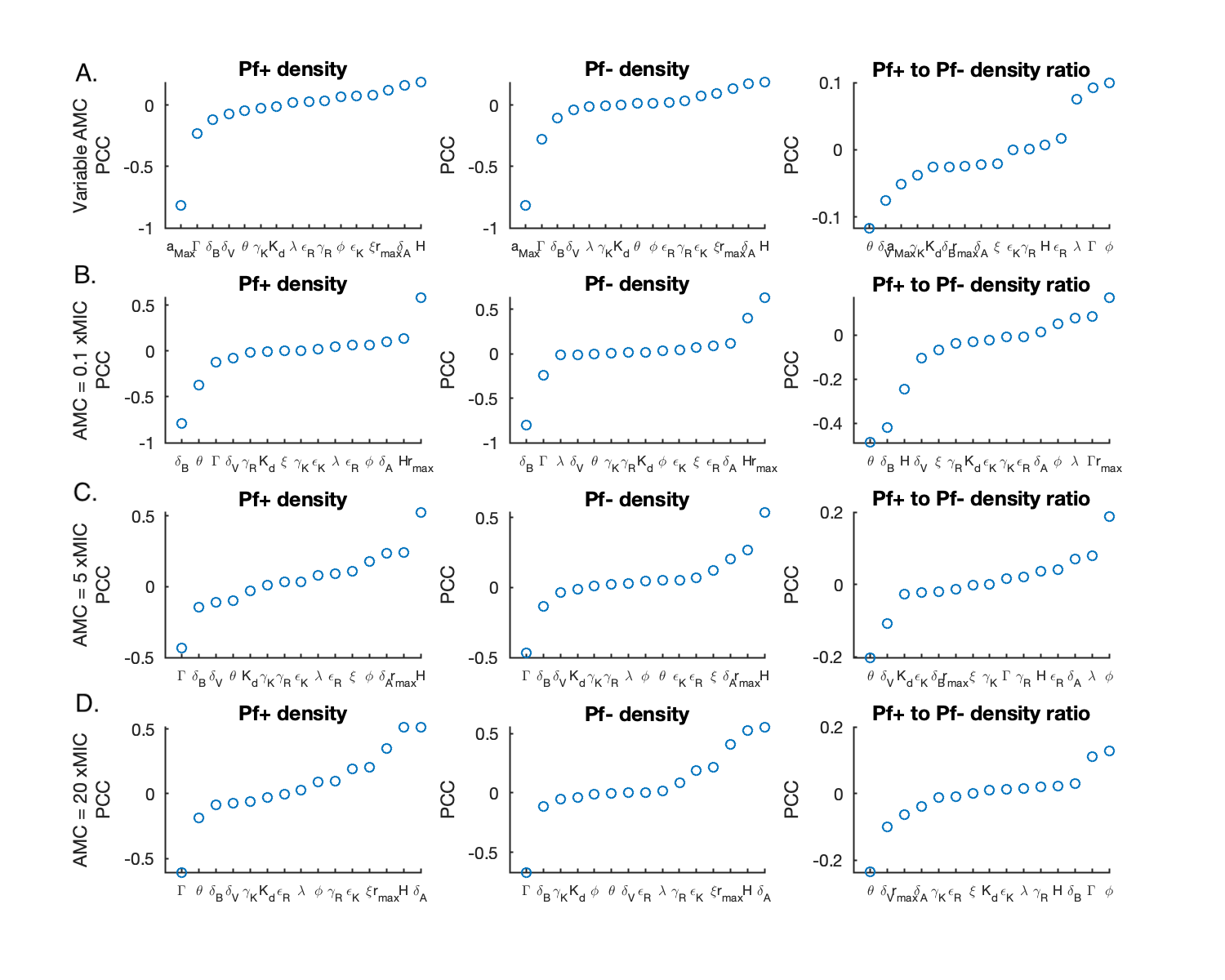

Supplement: FIG S1 [file msystems.00193-21-sf001.tif]
