# Supplementary material for: Filamentous Bacteriophages and the Competitive Interaction between Pseudomonas aeruginosa Strains under Antibiotic Treatment: a Modeling Study
Source: mSystems. 2021 Jun 22;6(3):e00193-21. doi: 10.1128/mSystems.00193-21 (PMC8269214; doi:10.1128/mSystems.00193-21)
Supplement: TABLE S1 [file msystems.00193-21-st001.docx]

|  | **Variable AMC** | | | **0.1 xMIC** | | | **5 xMIC** | | | **20 xMIC** | | |
| --- | --- | --- | --- | --- | --- | --- | --- | --- | --- | --- | --- | --- |
|  | **Pf+** | **Pf-** | **Ratio** | **Pf+** | **Pf-** | **Ratio** | **Pf+** | **Pf-** | **Ratio** | **Pf+** | **Pf-** | **Ratio** |
| $\boldsymbol{H}$ | *** | *** | - | *** | *** | *** | *** | *** | - | *** | *** | - |
| $\boldsymbol{r}_{\boldsymbol{max}}$ | *** | *** | - | *** | *** | *** | *** | *** | - | *** | *** | ** |
| $\boldsymbol{\Gamma}$ | *** | *** | *** | *** | *** | *** | *** | *** | - | *** | *** | *** |
| $\boldsymbol{\theta}$ | - | - | *** | *** | - | *** | *** | - | *** | *** | - | *** |
| $\boldsymbol{\epsilon}_{\boldsymbol{R}}$ | - | - | - | * | *** | - | *** | ** | - | - | - | - |
| $\boldsymbol{\gamma}_{\boldsymbol{R}}$ | - | - | - | - | - | - | - | - | - | *** | *** | - |
| $\boldsymbol{\phi}$ | ** | - | *** | * | - | - | *** | - | *** | *** | - | *** |
| $\boldsymbol{K}_{\boldsymbol{d}}$ | - | - | - | - | - | - | - | - | - | - | - | - |
| $\boldsymbol{\epsilon}_{\boldsymbol{k}}$ | ** | ** | - | - | - | - | - | * | - | *** | *** | - |
| $\boldsymbol{\xi}$ | ** | *** | - | - | ** | ** | *** | *** | - | *** | *** | - |
| $\boldsymbol{\gamma}_{\boldsymbol{k}}$ | - | - | - | - | - | - | - | - | - | * | * | - |
| $\boldsymbol{\delta}_{\boldsymbol{B}}$ | *** | *** | - | *** | *** | *** | *** | *** | - | *** | *** | - |
| $\boldsymbol{\delta}_{\boldsymbol{A}}$ | *** | *** | - | *** | *** | - | *** | *** | ** | *** | *** | - |
| $\boldsymbol{\lambda}$ | - | - | ** | - | - | ** | ** | - | ** | - | - | - |
| $\boldsymbol{\delta}_{\boldsymbol{V}}$ | ** | - | ** | ** | - | *** | *** | - | *** | ** | - | *** |
| $\boldsymbol{A}_{\boldsymbol{max}}$ | *** | *** | - | NA | NA | NA | NA | NA | NA | NA | NA | NA |
