# Supplementary material for: Filamentous Bacteriophages and the Competitive Interaction between Pseudomonas aeruginosa Strains under Antibiotic Treatment: a Modeling Study
Source: mSystems. 2021 Jun 22;6(3):e00193-21. doi: 10.1128/mSystems.00193-21 (PMC8269214; doi:10.1128/mSystems.00193-21)
Supplement: TABLE S2 [file msystems.00193-21-st002.docx]

| **Parameter** | **Parameter** | | **AMC** | | **Int. term** | | **Quad. term** | | **R^2^** |
| --- | --- | --- | --- | --- | --- | --- | --- | --- | --- |
|  | **Est.** | **Sig.** | **Est.** | **Sig.** | **Est.** | **Sig.** | **Est.** | **Sig.** |  |
| $\boldsymbol{\theta}$ | - 0.016 | *** | 0.19 | *** | 5.2 x 10^-3^ | *** | -0.022 | *** | 0.90 |
| $\boldsymbol{\phi}$ | 0.012 | *** | 0.21 | *** | - 1.0 x 10^-3^ | *** | -0.12 | *** | 0.83 |
| $\boldsymbol{\delta}_{\boldsymbol{V}}$ | -0.030 | *** | 0.15 | *** | 3.0 x 10^-3^ | *** | -0.12 | *** | 0.79 |
| $\boldsymbol{\delta}_{\boldsymbol{A}}$ | -0.014 | *** | 0.15 | *** | 5.6 x 10^-3^ | *** | -0.11 | *** | 0.92 |
| $\boldsymbol{\Gamma}$ | 8.5 x 10^-3^ | *** | 0.22 | *** | - 3.7 x 10^-3^ | *** | -0.12 | *** | 0.92 |
| $\boldsymbol{\epsilon}_{\boldsymbol{R}}$ | -2.2 x 10^-3^ | *** | 0.16 | *** | 1.6 x 10^-3^ | *** | -0.12 | *** | 0.92 |
| **Initial B** | 0.05 | *** | 0.19 | *** | - 8.8 x 10^-3^ | *** | -0.07 | *** | 0.89 |
